# Advantages of MabSelect VH3, a novel VH3-specific Protein A resin, over its two conventional counterparts in purifying a VHH-based trispecific antibody

**DOI:** 10.14440/jbm.0138

**Published:** 2025-09-19

**Authors:** Dongdong Fang, Wenjing Qi, Yifeng Li

**Affiliations:** Downstream Process Development, WuXi Biologics, Shanghai 200131, China

**Keywords:** Aggregate, Half-antibody, Homodimer, MabSelect VH3, Protein A, Trispecific antibody

## Abstract

**Background:**

Protein A affinity chromatography represents the most extensively used technique for initial product capture in antibody purification. The ligands of commercial Protein A resins from different, or even the same, vendors may exhibit distinct binding specificity. For example, MabSelect SuRe LX and MabSelect PrismA bind the antibody’s fragment crystallizable region and both the fragment crystallizable and variable heavy chain 3 (VH3) regions, respectively, while MabSelect VH3, a newly launched Protein A resin, binds the VH3 region exclusively.

**Objective:**

This study aimed to compare the capabilities of three Protein A resins—namely, MabSelect SuRe LX, MabSelect PrismA, and MabSelect VH3—in removing byproducts associated with a variable domain of heavy chain-only antibody-based trispecific antibody (TsAb).

**Methods:**

Clarified culture harvest containing the TsAb was processed separately using MabSelect SuRe LX, MabSelect PrismA, and MabSelect VH3 columns. For each column, byproduct separation was monitored by analyzing relevant elution fractions with sodium dodecyl sulfate-polyacrylamide gel electrophoresis and size-exclusion chromatography-high-performance liquid chromatography.

**Results:**

MabSelect VH3 demonstrated superior byproduct removal performance compared with the other two Protein A resins.

**Conclusion:**

MabSelect VH3 is particularly suitable for the purification of bispecific antibodies and TsAbs, where the product and byproducts contain different numbers of VH3 domains. For multispecific antibody purification, screening different affinity resins with distinct binding specificity is recommended, as this can help identify the most effective option for separation.

## 1. Introduction

Protein A affinity chromatography is the most widely used technique for product capture in antibody and fragment crystallizable (Fc)-fusion protein purification.^1^-^4^ Native Protein A is a cell wall component of *Staphylococcus aureus* that contains five homologous immunoglobulin (Ig) G-binding domains (designated E, D, A, B, and C).^5^-^8^ Protein A binds Igs at both the Fc region and the heavy-chain variable (VH) region belonging to the variable heavy chain 3 (VH3) family.^9^-^13^

Different commercial Protein A resins, even those from the same vendor, may exhibit distinct binding specificity due to their ligands’ unique origins and specific sequence modifications.^14^ For example, although MabSelect SuRe LX, MabSelect PrismA, and MabSelect VH3 are all manufactured by Cytiva, they bind the antibody’s Fc region, both the Fc and VH3 regions, and the VH3 region, respectively.^14^-^18^ The MabSelect SuRe LX ligand is based on the Z domain, which is derived from the B domain of native Protein A, and it possesses significantly reduced binding to VH3 domains.^14^,^15^ Therefore, MabSelect SuRe LX binds to the Fc region only. MabSelect PrismA was launched after MabSelect SuRe LX.^16^ While MabSelect PrismA ligand is also based on the Z domain and, on modification, exhibits enhanced binding affinity for VH3 domains.^16^,^17^ Thus, MabSelect PrismA can bind both Fc and VH3 regions. Although the MabSelect SuRe LX ligand is a tetramer of the engineered domain, the MabSelect PrismA ligand is constructed as a hexamer.^15^-^17^

MabSelect VH3 is a newly launched Protein A resin.^18^ It differs from all previously available Protein A resins (from Cytiva or other vendors) in that it shows affinity for the VH3 domain only. The MabSelect VH3 ligand consists of six identical subunits (*i.e*., engineered B domain of Protein A). We have previously demonstrated that this new Protein A resin, with its unique specificity, facilitates the separation of antibody species containing different numbers of VH3 domains.^19^ At a 6-min residence time, the dynamic binding capacity of MabSelect SuRe LX, MabSelect PrismA, and MabSelect VH3 at 10% breakthrough is approximately 60, 80, and 70 mg/mL, respectively.^15^,^16^,^18^

The variable domain of heavy chain-only antibody (VHH) is the smallest naturally occurring antigen-binding domain, and VHH domains have been utilized as building blocks for the construction of multispecific antibodies.^20^-^24^ Recently, in purifying a VHH-based trispecific antibody (TsAb), we observed that the culture harvest contained half-antibody, homodimer, and aggregates, in addition to the target TsAb, and a major difference between the product and byproducts was the number of VH3 domains that they contained. For affinity purification, separating the product from byproducts based on differences in binding valency is a general and effective strategy.^25^,^26^ As the three Protein A resins (*i.e*., MabSelect SuRe LX, MabSelect PrismA, and MabSelect VH3) possess distinct binding specificities toward the VH3 domain, we speculated that, in this TsAb case, they may present different capabilities for byproduct removal.

To test our hypothesis, clarified culture harvest containing this TsAb was processed separately using MabSelect SuRe LX, MabSelect PrismA, and MabSelect VH3 columns. As expected, due to MabSelect VH3’s non-Fc-binding feature and unique specificity for the VH3 domain, this Protein A resin achieved the most effective byproduct removal. MabSelect VH3 has emerged as an ideal tool for the purification of bispecific antibodies (bsAbs) and TsAbs, where the product and byproducts contain different numbers of VH3 domains.

In addition, the findings of the present study indicated that, for the same case, one Protein A resin may outperform the others due to its unique binding specificity. In fact, when combined with previous observations, this finding can be extrapolated to other affinity resins with distinct subdomain specificities (*i.e*., for a given case, one type of affinity resin may outperform the others). For example, when the product and byproducts differ in their numbers of kappa light-chain variable domains (Protein L binding motifs), Protein L-conjugated resins are a better choice for separation than other types of affinity resins.^25^ Therefore, in multispecific antibody purification, screening different affinity resins with distinct binding specificities is recommended to attain optimal separation.

## 2. Materials and methods

### 2.1. Materials

Ethanol, sodium acetate trihydrate (NaAc), sodium chloride (NaCl), sodium hydroxide, and tris(hydroxymethyl)aminomethane (Tris) were purchased from Merck, Germany. Acetic acid (HAc) was procured from J.T. Baker, USA. Precast SurePAGE 4–12% gradient Bis-Tris gels were bought from GenScript, China. Precision Plus Protein Unstained Standards were from Bio-Rad Laboratories, USA. 20X MES running buffer and 4X LDS sample buffer were purchased from Thermo Fisher Scientific, USA. MabSelect SuRe LX, MabSelect PrismA, and MabSelect VH3 resins came from Cytiva, Sweden. The BioCore SEC-300 stainless steel column (5 μm; 7.8 × 300 mm) was from NanoChrom, China. The target TsAb used in the present study was expressed in stably transfected CHO-K1 cells.

### 2.2. Equipment

An ÄKTA pure 150 system installed with Unicorn software version 7.8 (Cytiva, Sweden) was used for column chromatography. pH and conductivity were measured using a SevenExcellence S470 pH/conductivity meter (Mettler-Toledo, USA). Protein concentration was determined by employing a NanoDrop 2000 spectrophotometer (Thermo Fisher Scientific, USA). An Agilent 1260 liquid chromatography instrument (Agilent Technologies, USA) was utilized for size-exclusion chromatography-high-performance liquid chromatography (SEC-HPLC) analysis. A bioreactor system from Applikon Biotechnology, the Netherlands, was used for cell cultivation. The eStain LG protein staining system from GenScript, China, was employed for staining and destaining of protein gels.

### 2.3. Protein A Affinity chromatography

MabSelect SuRe LX, MabSelect PrismA, and MabSelect VH3 resins were individually packed in 0.5-cm-diameter columns with the bed height of 15.2, 14.8, and 15.4 cm, respectively. The column volumes (CVs) were approximately 3 mL. To compare the capability of these three Protein A resins in removing byproducts associated with the VHH-based TsAb, each column was loaded with clarified culture harvest at 30 mg of protein per mL of resin. After loading, the columns were washed consecutively with 50 mM Tris-HAc, 150 mM NaCl (pH 7.4), 50 mM NaAc-HAc, 1 M NaCl (pH 5.5), and 50 mM NaAc-HAc (pH 5.5), each for 5 CV.

After washing, the columns were eluted under a linear pH gradient, reaching 100% of 50 mM HAc at pH 3.0 over 20 CV. After identifying that MabSelect VH3 provided the most effective byproduct removal, a confirmation run was conducted using stepwise pH-gradient elution. For this run, the column was loaded at 13 mg of protein per mL of resin. After loading, the columns were washed consecutively with 50 mM Tris-HAc, 150 mM NaCl (pH 7.4), 50 mM NaAc-HAc, 1 M NaCl (pH 5.5), and 50 mM NaAc-HAc (pH 5.5), each for 5 CV. After washing, the column was eluted with 30 mM HAc (pH 3.9) for 5 CV.

For all runs, the system was operated at a flow rate of 180 cm/h (corresponding to a residence time of approximately 5 min).

### 2.4. SEC-HPLC

SEC-HPLC analysis of eluates from the three Protein A columns was performed using a BioCore SEC-300 stainless steel column (7.8 × 300 mm). According to the manufacturer’s application note, this column can be loaded up to 800 μg, and the typical injection amount ranges from 10 to 100 μg.^27^ The mobile phase consisted of 50 mM sodium phosphate and 300 mM NaCl, pH 6.8. After sample injection (100 μg per run), proteins were eluted isocratically at a flow rate of 1.0 mL/min for 20 min.

### 2.5. Non-reducing sodium dodecyl sulfate-polyacrylamide gel electrophoresis (SDS-PAGE)

Non-reducing SDS-PAGE was performed using a precast 4–12% gradient Bis-Tris gel from GenScript.^19^ In brief, protein samples were mixed with an appropriate amount of sample loading buffer and heated at 75°C for 5 min. After cooling, all samples were loaded at approximately 0.5 μg/well. Electrophoresis was conducted at 120 V for 90 min. The gel was stained and destained using the eStain L1 protein staining system from GenScript.

## 3. Results and discussion

### 3.1. The superior byproduct separation potential of MabSelect VH3 in the purification of a VHH-based TsAb

**Figure 1:**
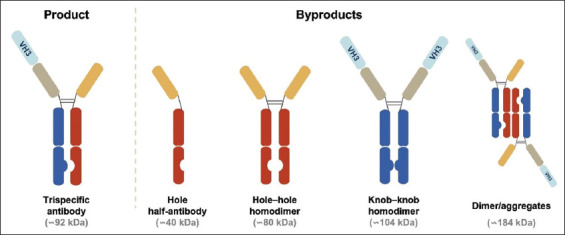
Schematic representation of the target TsAb and major byproducts. The target TsAb contains three distinct VHH domains, and one of the two VHH motifs in the knob heavy chain belongs to the VH3 family (labelled). Heavy-chain heterodimerization is facilitated using the knobs-into-holes technology. In addition to the target TsAb, the culture harvest contained significant amounts of hole half-antibody, hole–hole homodimer, knob–knob homodimer, and aggregates. A dimer is shown as a representative of aggregates, although higher-order aggregates are also present. Approximate molecular weight is indicated for each species. Abbreviations: VH3: Variable heavy chain 3; VHH: Variable domain of heavy chain-only antibody; TsAb: Trispecific antibody.

In purifying a VHH-based TsAb, which employed knobs-into-holes technology to promote heavy-chain heterodimerization, we observed that the culture harvest contained several product-related byproducts, including hole half-antibodies, homodimers (hole–hole and knob–knob), and aggregates (Figure 1), in addition to the target TsAb. Thanks to the unique design of this TsAb, a major difference between the product and byproducts is the number of VH3 domains they contain. For example, while the target TsAb contains one VH3, the hole half-antibody and hole–hole homodimer contain none, and the knob–knob homodimer contains two VH3 domains (Figure 1). Aggregates of the target TsAb can contain two or more VH3 domains, depending on the number of copies present. These findings suggest that Protein A resins with different VH3 binding specificities may exhibit distinct byproduct separation potential. To test this hypothesis, we processed the TsAb-containing culture harvest separately using MabSelect SuRe LX, MabSelect PrismA, and MabSelect VH3 columns, thereby allowing for a parallel comparison of the byproduct-removal potential of these three Protein A resins.

**Figure 2:**
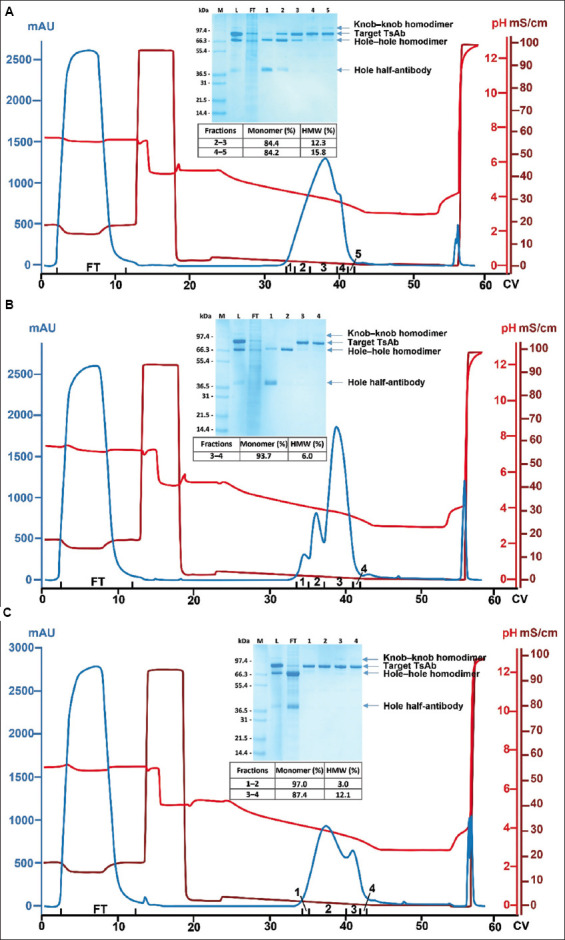
Chromatograms of MabSelect SuRe LX (A), MabSelect PrismA (B), and MabSelect VH3 columns (C). The load material was clarified culture harvest containing the target TsAb and byproducts, as shown in Figure 1. All runs were performed under linear pH gradient elution. Insets show the corresponding SDS-PAGE analyses, with lanes 1–4/5 representing elution fractions 1–4/5. Bands corresponding to the target TsAb, knob–knob homodimer, hole–hole homodimer, and hole half-antibody are indicated. Insets also contain relevant SEC-HPLC data. Abbreviations: FT: Flow-through; HMW: High-molecular-weight species; L: Load; M: Protein markers; SDS-PAGE: Sodium dodecyl sulfate-polyacrylamide gel electrophoresis; TsAb: Trispecific antibody.

For each of the three columns (MabSelect SuRe LX, MabSelect PrismA, and MabSelect VH3), elution was performed under a linear pH gradient, and the corresponding chromatograms are illustrated in Figure 2. As shown in Figure 2A, MabSelect SuRe LX achieved poor resolution. The elution profile contained a single peak with a small shoulder. According to the SDS-PAGE and SEC-HPLC data (Figure 2A), the hole half-antibody/hole–hole homodimer and knob–knob homodimer/aggregates were only slightly enriched in the early and late elution fractions, respectively. In the main peak portion (fractions 2 and 3), the target TsAb was contaminated with a considerable amount of hole–hole homodimer (Figure 2A, inset). As MabSelect SuRe LX binds the Fc region alone, and because the heterodimer and both types of homodimers contain a similar Fc region, it is expected that this Protein A resin provides poor resolution between the heterodimer and homodimers. In addition, the data are consistent with our previous findings that, under regular conditions, traditional Protein A resins showed limited and poor capabilities for half-antibody and aggregate separation, respectively.^28^,^29^

MabSelect PrismA accomplished greater separation than MabSelect SuRe LX, as reflected in the corresponding chromatogram (Figure 2B) and associated analytical data (Figure 2B, inset). The elution profile demonstrated three relatively well-resolved peaks, which mainly corresponded to hole half-antibody, hole–hole homodimer, and the target TsAb, respectively. A major difference between MabSelect SuRe LX and MabSelect PrismA was the enhanced VH3 binding of the latter. Notably, MabSelect PrismA’s VH3 binding capability contributed to the improved resolution. Among the three species (*i.e*., hole half-antibody, hole–hole homodimer, and the target TsAb), the hole half-antibody, which contains half of the Fc domain and no VH3 domain, showed the weakest binding. However, compared with MabSelect SuRe LX, MabSelect PrismA’s VH3 binding capability improved the resolution between the hole half-antibody and the target TsAb. While both the hole–hole homodimer and the target TsAb contain an intact Fc domain, the TsAb exhibited stronger binding affinity due to the presence of a VH3 domain.

Although MabSelect PrismA effectively separated the hole half-antibody and hole–hole homodimer, the main peak (fractions 2 and 3) was contaminated with the knob–knob homodimer (according to the SDS-PAGE results) and 6% aggregates (according to the SEC-HPLC data). The knob–knob homodimer contains two VH3 domains, whereas the target heterodimer contains only one. However, this difference was not sufficient for the complete separation of these two species. The poor aggregate removal is consistent with our earlier observations—traditional Protein A resins generally lack the capability to separate aggregates under typical pH gradient elution.^29^

When the culture harvest was processed on the MabSelect VH3 column, the hole half-antibody and hole–hole homodimer, which do not contain a VH3 domain, did not bind and appeared in the flow-through (Figure 2C, inset). The knob–knob homodimer and aggregates, which contain two and multiple VH3 domains, respectively, bound more tightly than the target TsAb—which contains only one VH3 domain—and therefore were enriched in the shoulder peak at the end of the elution (Figure 2C). Consequently, the main elution fractions (fractions 1 and 2) contained a relatively pure monomer of the target TsAb (Figure 2C, inset). Compared with MabSelect SuRe LX and MabSelect PrismA, MabSelect VH3 provided the best byproduct separation. In certain cases, however, VH3 binding can be a disadvantage. For example, in the purification of bsAbs based on the asymmetric Protein A binding design, resolution between the target Fc-Fc* heterodimer (*i.e*., one Fc contains a mutation that ablates its Protein A binding capability) and Fc*-Fc* homodimer (*i.e*., both Fc regions contain Protein A binding-disruption mutations) may be compromised if a Protein A resin with VH3 binding is used.^30^-^32^ Nevertheless, in this study, because the primary distinguishing feature between the target TsAb and byproducts is the number of VH3 domains they contain, VH3 binding is clearly an advantage.

### 3.2. Confirmation of MabSelect VH3’s strong aggregate separation capability

Unlike traditional Protein A resins, which lack the capability to separate co-binding aggregates, MabSelect VH3 has been shown to exhibit improved aggregate separation performance.^19^ To further confirm MabSelect VH3’s superiority in aggregate removal potential, in addition to its half-antibody and homodimer removal capability, a run was conducted under stepwise pH gradient elution using the same TsAb-containing culture harvest, which contained approximately 12% aggregates according to SEC-HPLC analysis (profile not shown).

**Figure 3:**
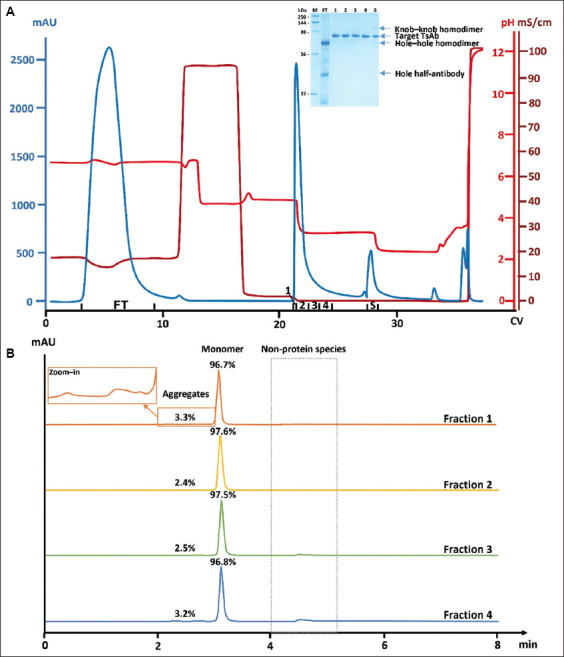
Purification and analytical data of a TsAb-containing culture harvest processed using MabSelect VH3 column. (A) Chromatogram of a MabSelect VH3 run conducted under stepwise pH gradient elution. The load material was the same as that used for the linear pH gradient elution runs. The inset shows the corresponding SDS-PAGE analysis of the relevant fractions, with lanes 1–4/5 representing elution fractions 1–4/5. Bands corresponding to the target TsAb, knob–knob homodimer, hole–hole homodimer, and hole half-antibody are indicated. (B) SEC-HPLC profiles of sample aliquots from elution fractions 1–4. Percentages of monomer and aggregates are indicated. For fraction 1, a zoomed view of the aggregate peaks is provided. Abbreviations: FT: Flow-through; HMW: High-molecular-weight species; L: Load; M: Protein markers; SDS-PAGE: Sodium dodecyl sulfate-polyacrylamide gel electrophoresis; SEC-HPLC: Size-exclusion chromatography-high-performance liquid chromatography; TsAb: Trispecific antibody.

The chromatogram of this run is presented in Figure 3A. In addition to good clearance of hole half-antibody and homodimers (both hole–hole and knob–knob), as suggested by the SDS-PAGE results (Figure 3A, inset), effective aggregate removal was indicated by SEC-HPLC analysis of the four elution fractions (fractions 1–4), whose monomer content reached 96.7%, 97.6%, 97.5%, and 96.8%, respectively (Figure 3B for the SEC-HPLC profiles). Thus, the results confirmed MabSelect VH3’s strong aggregate separation capability. According to the SDS-PAGE results (Figure 3A, inset), the knob–knob homodimer was predominantly in the strip. As aggregates and knob–knob homodimer were previously found in the same fraction under linear pH gradient elution (Figure 2C), these findings indicate that aggregates were also in the strip with knob–knob homodimer under the stepwise pH gradient elution. However, this cannot be demonstrated by SDS-PAGE analysis of the strip, since under denaturing conditions, aggregates dissociated into monomers. Nevertheless, the SEC-HPLC results provided convincing evidence for successful aggregate removal.

## 4. Conclusion

Different Protein A resins, even from the same vendor, can exhibit distinct binding specificities. In this study, we compared three Protein A resins from Cytiva, namely, MabSelect SuRe LX, MabSelect PrismA, and MabSelect VH3, in terms of their capability to remove byproducts associated with a VHH-based TsAb. While MabSelect SuRe LX and MabSelect VH3 bind to the antibody Fc region and VH3 region, respectively, MabSelect PrismA can bind to both regions. A major difference between the target TsAb and its byproducts was the number of VH3 domains they contained, and MabSelect VH3 provided the most effective byproduct removal. Thus, VH3’s unique binding specificity renders it an ideal tool for the purification of bsAbs and TsAbs, where the product and byproducts differ in the number of VH3 domains. However, it should be noted that although MabSelect VH3 demonstrates strong capabilities in separating byproducts and aggregates, its application is limited to VH3-containing antibodies and antibody fragments with a VH3 domain recognized by MabSelect VH3.

In multispecific antibody purification, affinity media with distinct subdomain specificities—including Protein A resins and other commercial resins that selectively bind the variable or constant regions of the light chain, the CH1 region of the heavy chain, or the CH3 region of the heavy chain—can yield markedly different outcomes. Separation of byproducts from the product based on differences in binding valency is an effective strategy. However, a prerequisite for this approach is the identification of an affinity medium for which the product and byproducts differ in binding valency. Therefore, it is highly recommended to carefully evaluate the platform on which the multispecific antibody is constructed to determine whether the product and potential byproducts differ in the number of any binding motifs. Identifying such differences enables the rapid selection of the most effective affinity medium for purification.

## Data Availability

The data and supporting information are available either within the article or on request.
